# Biomarkers for Cancer Cachexia: A Mini Review

**DOI:** 10.3390/ijms22094501

**Published:** 2021-04-26

**Authors:** Zhipeng Cao, Kening Zhao, Irvin Jose, Nick J. Hoogenraad, Laura D. Osellame

**Affiliations:** 1 Department of Biochemistry and Genetics, La Trobe University, Bundoora, VIC 3086, Australia; K.Zhao@latrobe.edu.au (K.Z.); I.Jose@latrobe.edu.au (I.J.); N.Hoogenraad@latrobe.edu.au (N.J.H.); 2 Department of Laboratory Medicine, Nanfang Hospital, Southern Medical University, Guangzhou 510515, China; 3 Tumour Targeting Laboratory, Olivia Newton-John Cancer Research Institute, School of Cancer Medicine, La Trobe University, Melbourne, VIC 3084, Australia

**Keywords:** cancer cachexia, biomarkers, cachexia-inducing factors

## Abstract

Cancer cachexia is a common condition in many cancer patients, particularly those with advanced disease. Cancer cachexia patients are generally less tolerant to chemotherapies and radiotherapies, largely limiting their treatment options. While the search for treatments of this condition are ongoing, standards for the efficacy of treatments have yet to be developed. Current diagnostic criteria for cancer cachexia are primarily based on loss of body mass and muscle function. However, these criteria are rather limiting, and in time, when weight loss is noticeable, it may be too late for treatment. Consequently, biomarkers for cancer cachexia would be valuable adjuncts to current diagnostic criteria, and for assessing potential treatments. Using high throughput methods such as “omics approaches”, a plethora of potential biomarkers have been identified. This article reviews and summarizes current studies of biomarkers for cancer cachexia.

## 1. Introduction

Cancer cachexia is a wasting condition, mainly characterized by loss of muscle and adipose tissue. This condition severely limits the quality of life and use of cancer therapies. Cancer cachexia is mainly associated with cancers caused by solid tumors and may affect up to 80% of patients with late-stage cancer, and be responsible for more than 20% of cancer-related deaths [1]. Despite extensive association with cancer morbidity and mortality, no effective interventions for cancer cachexia have been developed. To understand this condition, several cachexia-inducing factors have been identified in animal models over the last decade. However, few of these targets have been evaluated in clinical settings. Unlike anti-cancer treatments, studies for treating clinical cancer cachexia are limited due to the lack of explicit criteria to assess both the effects of treatment and endpoints of clinical trials [2]. Another limitation is that cancer cachexia produced by different types of cancers may have different causes. Furthermore, the symptoms of cachexia such as loss of muscle mass may result from other causes such as lack of muscle use or deficient nutrition. Most clinical studies have recruited patients with a single type of cancer, which lowers the applicability of these biomarkers for cachexia induced by other cancers. A standardized evaluation is not only necessary to analyze the clinical trial itself, but also beneficial for comparing treatments in different trials. A discrepancy in assessing cancer cachexia has been seen within the same study performed in different countries. For instance, the U.S. Food and Drug Administration and the European Medicines Agency used different assessment endpoints for the same POWER 1 and 2 trial that investigated the function of a selective androgen receptor modulator in non-small cell lung cancer patients with muscle loss [3,4].

Current meaningful endpoints for cancer cachexia studies are divided into gravimetric and functional endpoints. A gravimetric endpoint is simply based on the change of muscle mass or lean body mass (LBM), while a functional endpoint is evaluated by a change in physical function such as improved stair climbing power and hand grip strength [5]. In the POWER (enobosarm) and ROMANA (anamorelin) clinical trials, increased lean body mass was achieved with these treatments, whilst improvement in muscle function was not significant [3,4,6]. Therefore, it is difficult to conclude whether these treatments were effective. On the other hand, a positive correlation between muscle mass and strength was shown in the general population, but not in the elderly [7,8]. Taking this into consideration, the age of the recruited patients could be a significant factor affecting the results of these clinical trials.

A biomarker-based evaluation system for cancer cachexia could solve these issues as biomarkers are a more objective and reliable diagnostic tool than functional assessment. Several potential biomarkers have so far been identified including some cachexia-inducing factors [9,10], pro-inflammatory cytokines [11,12], lipids [13], protein and fat degradation products [14], and microRNAs [15]. Nevertheless, none of these candidates have been widely validated and accepted as clinical diagnostic standards for cancer cachexia. We hereby summarize the potential biomarkers for cancer cachexia that have been evaluated in clinical studies to gain a better understanding of these potential biomarkers and directing future studies in the field. Some biomarkers of cancer cachexia were reviewed by Loumaye et al. [16], mainly focusing on the findings of these biomarkers. This review, in contrast, not only includes more recent discoveries of biomarkers for cancer cachexia, but also summarizes important details such as circulating levels of these biomarkers in certain cancer types, giving reference for future clinical studies of cancer cachexia.

## 2. Cachexia-Inducing Factors

Many cachexia-inducing factors such as activin A (ActA) [17], myostatin (Mstn) [18], and growth/differentiation factor 15 (GDF15) [19] are members of the transforming growth factor (TGF)-β family, capable of inducing muscle wasting and fat loss in preclinical cancer cachexia models. The mechanism by which ActA and Mstn induce muscle wasting in cancer cachexia are well understood. ActA and Mstn bind to the type IIB activin receptor (ActRIIB) on the muscle cell membrane, leading to the activation of multiple signaling pathways and subsequently increasing expression of two E3 ubiquitin ligases, Muscle RING-finger protein-1 (MuRF1) and Muscle Atrophy F-box gene (Atrogin-1) [20]. MuRF1 and Atrogin-1 accelerate protein degradation in muscle cells through the proteasome to cause muscle wasting [20]. High circulating levels of ActA were found in cancer cachexia animal models [21] and patients [22] in whom this was associated with poor survival of patients with various types of cancer [23,24,25]. A positive correlation of ActA and cachexia has been shown in patients with non–small-cell lung cancer (NSCLC), malignant pleural mesothelioma (MPM), pancreatic ductal adenocarcinoma (PDAC), and colorectal cancer [22,26,27]. Mstn, in contrast, is not elevated in the C-26 murine cancer cachexia model [28], though it functions through the same receptor as ActA. Interestingly, some studies have found that cancer patients with weight loss had lower levels of circulating Mstn [22,29], despite it being a cachexia-inducing factor. GDF15, a recently identified cachexia-inducing factor, was shown to be a promising target for treating cancer cachexia [30]. GDF15 induces cancer cachexia via its action on the hypothalamus to decrease appetite [31] and elicits a lipolytic response in adipose tissue [30]. High circulating levels of GDF15 are associated with weight loss and shorter survival in cancer patients [32,33,34]. However, association between GDF15 level and weight loss was only confirmed in patients with lung but not pancreatic cancer [33]. Additionally, another study found no difference in GDF15 levels between cancer patients with weight loss and those without weight change [35]. This study demonstrated an association between GDF15 levels and anorexia, suggesting that it could be an biomarker for anorexia rather than cachexia [35].

Zinc-α2-glycoprotein (ZAG) is a lipid mobilizing factor that can be secreted by both tumors and host tissues. Enhanced ZAG production was observed in adipose tissue from cachectic mice and patients [36,37]. A further study found that ZAG promotes white adipose tissue (WAT) browning and energy wasting through the expression of peroxisome proliferator–activated receptor γ (PPARγ), PPARγ coactivator 1α, and early B cell factor 2 [38]. Elevation of circulating ZAG levels was shown to correlate with cachexia in pancreatic cancer patients [39]. However, other studies have suggested no significant differences in circulating ZAG levels between cachectic and weight stable cancer patients [37,40]. 

Fibroblast growth factor 21 (FGF21), a hormone regulating several metabolic pathways, is associated with brown adipose tissue (BAT) activity in humans [41,42], although the detailed mechanism is unknown. Mice with muscle-specific FGF21 deletion were resistant to muscle atrophy and overexpression of FGF21 in muscle was sufficient to induce muscle loss by 15% [43]. Autophagy was shown to be induced by FGF21 in muscle, which could be responsible for muscle atrophy [43]. Although the involvement of FGF21 in cancer cachexia needs to be further investigated, a clinical study has confirmed a correlation of FGF21 with cachexia in elderly patients [44]. Association of serum FGF21 in sarcopenia, a condition associated with loss of muscle activity in older adults, was also confirmed [45]. Correlation of serum FGF21 in cancer-induced cachexia requires further investigation.

Parathyroid Hormone release Peptide (PTHrP) was shown to promote browning of fat in a cancer cachexia mouse model [46]. In mice, association of circulating PTHrP with cancer cachexia is controversial even for cachexia induced by the same cancer cell line [46,47]. Conditioned medium from Lewis Lung Carcinoma (LLC) was shown to stimulate expression of thermogenic genes in fat cells and increased plasma PTHrP levels were observed in mice bearing LLC tumors [46]. However, another study found no abnormal secretion of PTHrP from LLC cells compared with non-tumorigenic cell lines and a tumor cell line that does induce cachexia [47]. There was no significant difference in circulating PTHrP levels in mice bearing LLC tumors and control mice [31]. High levels of circulating PTHrP was shown to be associated with lower lean body mass and higher resting energy expenditure in patients with lung and colorectal cancer [46,48]. 

To identify blood-based biomarkers for cancer cachexia induced by all types of cancer, a study was performed to profile plasma cytokines and mRNA in blood, which included patients with breast, upper gastrointestinal, lung, hepatobiliary, pancreatic, prostate, colorectal, head and neck cancers [49]. Elevated circulating angiotensin II (Ang II) and increased mRNA expression of neutrophil-derived proteases (NDPs) were observed in pre-cachectic (no cachexia but high C-reactive protein level) and cachectic patients [49]. Ang II was shown to promote proteolysis and apoptosis by downregulating phosphor-Akt and activating caspase-3 in skeletal muscle [50]. Additionally, Ang II can increase expression of cytokines such as IL-6, TNF-α, and glucocorticoids and acts on hypothalamic neurons to reduce appetite [51]. NDP such as cathepsin B and G was shown to release Ang I and Ang II from angiotensinogen [52,53]. Therefore, both Ang II and NDP are not only promising biomarkers, but also therapeutic targets for cancer cachexia.

Overall, despite extensive studies on the mechanisms of cachexia-inducing factors, more studies are required to investigate their utility as biomarkers for cancer cachexia (Table 1). Additionally, identification of other cachexia-inducing factors will provide more candidates for biomarkers of cancer cachexia. A recent study established two reporter cell lines to detect potential factors inducing muscle wasting [54], which could be advantageous to rapidly identify other cachexia-inducing factors that directly induce muscle wasting.

## 3. Inflammatory Factors

Many inflammatory cytokines are important players in cancer cachexia and can be released by both host tissues and tumors. Upregulation of these cytokines have been observed in several preclinical cancer cachexia models [17,21,55]. Correlation between these cytokines and cancer cachexia is controversial in humans. An early study found a negative correlation between serum TNF-α levels and body weight and body mass index in pancreatic cancer patients [56]. Another study showed that circulating levels of TNF-α were not associated with increased survival of patients with advanced cancer [57]. It should be noticed that TNF-α has a short half-life (18.2 min) and low bioavailability [58], marking it difficult to measure.

Among all the inflammatory cytokines related to cancer, IL-6 is one of the more promising biomarkers for cachexia. Circulating IL-6 levels showed positive correlation to both survival and lean body mass in patients with advanced lung cancer [57,59]. IL-6 levels were also positively correlated with both tumor stage and weight loss in gastrointestinal cancer patients [60]. Nevertheless, another clinical study for non-small lung cancer patients suggested no significant difference in circulating levels of TNF-α and IL-6 between cachectic and non-cachectic patients [61]. Compared with IL-6, IL-1β is considered a promising biomarker that associates with clinical features of cachectic conditions in patients with gastrointestinal and lung cancer [62]. Serum IL-8 level was also elevated in cachectic patients with pancreatic cancer compared with weight stable cancer patients [63]. 

Monocyte chemoattractant protein-1 (MCP-1) is a factor that recruits inflammatory cells during infection [64]. Mice with MCP-1 deletion showed attenuated bone loss in the LLC cancer cachexia model [65]. Circulating level of MCP-1 was increased in the C26 model [66], suggesting that it could be a biomarker for cancer cachexia. In a comprehensive study that tested levels of 25 circulating factors such as TNF-α, IFN-γ, IL-1β, and IL-6 in cachectic and weight stable patients, monocyte chemoattractant protein-1 (MCP-1) was the only biomarker significantly increased in cachectic patients with pancreatic cancer [11]. Circulating levels of MCP-1 in cachexia induced by other cancers does not appear to have been evaluated.

C-reactive protein (CRP) is a plasma protein that serves as an indicator for inflammation [67]. Increased circulating levels of CRP have been found in cancer cachexia patients compared with weight stable cancer patients and non-cancer patients [60,68,69,70]. Although circulating CRP has been widely assessed in many studies, these are difficult to compare as there are differences in the CRP cut-off value among these studies [71]. Decreased albumin level was reported in patients with cancer cachexia compared with weight stable cancer patients or non-cancer controls [70], and in another study, it was shown to negatively correlate with CRP levels [72]. 

Collectively, evidence shows the potential of inflammatory cytokines as biomarkers for cancer cachexia, however, none of these are elevated in cachectic patients across multiple types of cancer (Table 2). Further studies are required to assess the levels of these cytokines in a large cohort of cachectic patients encompassing different cancers. It should also be noted that these inflammatory factors could be elevated in other conditions such as infection. An additional differentiation of cancer cachexia from other inflammatory diseases may be required if using these factors in diagnosing cancer cachexia.

## 4. Muscle and Fat Wasting Products

Mechanisms of muscle wasting in cancer cachexia have been widely studied over the past decade. Increased protein degradation and decreased protein synthesis are considered as the main causes of muscle loss during cancer cachexia. Molecular mechanisms of muscle atrophy in cancer cachexia have been reviewed by Wei et al. [74]. From a metabolic view, several dysfunctional states are developed during cancer cachexia, out of which insulin resistance is commonly observed in patients. The mechanism of how insulin resistance contributes to cachectic symptoms is reviewed by Porporato [75]. Insulin resistance induces muscle wasting by increasing protein breakdown and decreasing protein synthesis. After protein degradation, some products are released into circulation, making them potential biomarkers for monitoring muscle wasting in cancer cachexia (Table 3).

Collagen is one of the main components of the extracellular matrix of skeletal muscle and is degraded during muscle atrophy [80,81]. As collagen is the target of different proteases, a variety of fragments of collagen are generated by proteases. In a human study that evaluated the feasibility of using collagen fragments as serological biomarkers of lean body mass, increased levels of collagen VI fragment-C6M was found in patients with head and neck squamous cell carcinoma compared with healthy controls [14]. However, C6M levels only correlated with LBM in healthy controls but not in recovered cancer patients [14]. Further investigation is required to find if the level of circulating collagen specifically correlates to muscle wasting in cancer cachexia. Using mass spectrometry, muscle wasting products including myosin species, α-spectrin, nischarin, microtubule-actin crosslinking factor, microtubule-associate protein-1B, and bullous pemphigoid antigen 1 were found in urine samples from cachectic patients [82]. However, this finding is based on a small cohort of patients and requires analysis in a larger sample size prior to clinical application.

Some biomarkers may remain in the muscle itself and are not released into circulation. For example, β-dystroglycan was shown to be increased in skeletal muscle of cachectic patients with upper gastrointestinal cancer [69]. Muscle Ca^2+^/calmodulin (CaM)-dependent protein kinase II (CaMKII) β and tyrosine kinase with immunoglobulin-like and EGF-like domains 1 (TIE1) were also positively associated with weight loss in upper gastrointestinal cancer patients [83]. However, these results were only supported by western blot and RNA analysis of muscle samples. The drawback of an intramuscular biomarker is the requirement for an invasive muscle biopsy in cachectic patients.

Loss of adipose tissue is another common symptom observed in cancer cachexia. The adipose tissue plays a vital role even in cancer with a relatively low cachexia rate [84]. Browning and atrophy of mammary fat were found in patients with breast cancer [85]. Crosstalk between muscle and adipose tissue has been shown in cancer cachexia [86,87]. Brown adipose tissue was shown to induce muscle atrophy via secretion of myostatin [87], while muscle-derived IL-15 was able to modulate adipose tissue deposition [88]. During fat loss, lipids are degraded into glycerol and fatty acids by the action of lipolytic enzymes such as hormone-sensitive lipase and adipose triglyceride lipase [89]. Elevated circulating levels of both glycerol and fatty acids have been observed in cachectic patients with gastrointestinal cancer compared with weight stable cancer patients [76,77,78,90]. Further studies are required to test whether these can serve as biomarkers for cachexia induced by other cancers.

In a study investigating lipid profiles of cancer cachexia, increased circulating levels of sphingolipids were found in different preclinical models and cachectic patients with gastrointestinal cancers [13]. Specifically, circulating levels of hexosyl-ceramides (HCERs) and lactosyl-ceramides (LCERs) were significantly higher in cachectic patients compared with weight stable patients [13]. Although this study was limited by the small cohort of patients (20 cachectic and 19 weight stable cancer patients), the finding was promising, as it shows a consistency between preclinical models and clinical patients. 

## 5. MicroRNAs

MicroRNAs are a class of small non-coding RNA of about 17–22 nucleotides in length, which have been found in a wide range of biological processes. The main role of microRNAs is to regulate gene expression. There is a growing body of evidence detailing the emerging role of microRNAs and other non-coding RNAs in cancer cachexia, which is reviewed by Santo et al. [91]. Many of these RNAs function in target organs such as skeletal muscle and adipose tissue. Hereby, we summarized several microRNAs that were detected in serum or plasma and are related to cancer cachexia (Table 4).

MicroRNA-21 is one of the well-studied oncogenic microRNAs that targets several tumor suppressor genes [95]. Expression of microRNA-21 is upregulated in many hematological and non-hematological malignancies [95]. Microvesicles containing increased levels of microRNA-21 were secreted by lung and pancreatic tumors, and shown to induce apoptosis of skeletal muscle cells [96]. Expression of serum microRNA-21 was significantly elevated in colorectal cancer patients with a low muscle mass index [92]. These findings support the feasibility of microRNA-21 as a biomarker for cancer cachexia.

Another similar study demonstrated that circulating microRNA-203 levels were upregulated in cachectic patients with colorectal cancer [93]. Elevated serum microRNA-203 was deemed an independent risk factor for myopenia in these patients [93]. Moreover, Baculoviral IAP Repeat Containing 5, a member of the apoptosis inhibitor family, was identified as the target of microRNA-203 in skeletal muscle [93], suggesting that circulating microRNA-203 could lead to muscle wasting by inducing apoptosis of muscle cells. Circulating levels microRNA-203 were also shown as a predictive biomarker for prognosis and metastasis in colorectal cancer patients [97].

A study measuring circulating microRNA-130a levels in head and neck cancer patients found that low microRNA-130a was associated with high circulating TNF-α levels, and patients with low circulating microRNA-130a expression had a higher risk of weight loss [94]. Nutritional assessment using subjective global assessment was also significantly improved by combining analysis of circulating microRNA-130a levels, particularly in cancer patients with more than 5% weight loss [94]. 

More comprehensive studies may identify other potential non-coding RNAs that may be used as a diagnostic tool for cancer cachexia. One of the most attractive characteristics of these non-coding RNAs is that they are generally secreted into circulation via microvesicles such as exosomes, ensuring stability during the processing of blood samples.

## 6. Other Potential Biomarkers

There are several other factors that are also differentially expressed in cachectic patients compared to weight stable controls (Table 5). A study involving gastrointestinal cancer patients with and without cachexia showed that the plasma level of carnosine dipeptidase 1 (CNDP1) is significantly lower in cachexia patients [98]. Apoliprotein A4 and Dachshund Family Transcription Factor 1 were also reduced in cachectic patients compared with weight stable cancer patients [98]. Additionally, this study found that circulating levels of Asparaginyl-tRNA Synthetase 2, ATPase type 13A4, and B-cell CLL/Lymphoma 3 were upregulated and may be used as biomarkers for cancer cachexia [98]. The functions of these factors in cancer cachexia have not been investigated.

Tissue inhibitor of metalloproteinases-1 (TIMP-1) is involved in tumorigenesis [100], cancer progression [101], tumor growth [102], and apoptosis [103]. Circulating levels of TIMP-1 are upregulated in different cancer types such as gastric [104], lung [105], breast [106], colorectal [107], and pancreatic cancer [108]. In a study investigating the association of plasma TIMP-1 level with cachexia in pancreatic cancer patients, elevated TIMP-1 levels were found in patients with weight loss and without jaundice [99]. However, in another study using an aptamer-based discovery platform to identify serum protein biomarkers for pancreatic cancer cachexia, TIMP-1 was only associated with stages of cancer but not weight loss [109]. Previously identified biomarkers such as GDF15 did not show significant correlation with weight loss in pancreatic cancer patients in this study [109].

## 7. Conclusions

Although many promising biomarkers for cancer cachexia have been identified, none are yet approved for clinical use. Some potential biomarkers were shown to be adequate indicators of cachexia induced by some cancers, but not consistent across a range of cancer types. Consequently, multiple biomarkers may be required to predict and monitor progression of this condition in the general cancer population. A recent study found expression profiles of 12 cachexia-inducing factors correlate with the prevalence of cachexia and weight loss in different cancer types [110]. Cancers with higher cachexia incidence were shown to have elevated serum levels of these factors [110]. This suggests that establishing an assessment standard involving several potential biomarkers may be more suitable for predicting and monitoring cancer cachexia than using a single biomarker. However, whether the levels of these factors correlate with the severity of weight loss has not yet been investigated. Not only is predicting cachexia important, being able to monitor the progression of the condition is also essential. Microbial dysbiosis has been observed in preclinical cancer cachexia models [111,112], which could be a potential diagnostic reference for cancer cachexia. *Enterobacteriaceae* levels were increased, while a reduction of *lachnospiraceae* and *ruminococcaceae* were found in C26 cancer cachexia model [111,112]. Such alteration has not been confirmed in human cancer cachexia.

Interestingly, recent studies have shown that body composition changes, especially muscle alteration, differ by gender in cancer patients [113,114]. Another study found that female and male mice had differences on body weight flux, specific force, protein concentration, and muscle mass in a preclinical cancer cachexia model [115]. Hence, some biomarkers could be gender specific and may not be suitable for whole cancer cachexia population. Chemotherapy is the primary treatment for most cancers, which could lead to development of cachexia, as it is a highly invasive and uncomfortable treatment designed to aggressively treat the cancer, impinging on many aspects of patient lifestyle and wellbeing [116,117]. Nonetheless, mechanisms of how chemotherapy drugs induce cachexia remain unknown. It is expected that different chemotherapy treatments may have distinct effects on cachexia, thus altering levels of some biomarkers. Future study should focus on pinpointing the correlation of various chemotherapy drugs and potential biomarkers for cancer cachexia.

Although utilization of these biomarkers provides potential diagnosis of cancer cachexia, many of these could also be promising targets for treating this condition [10]. As improvements in both muscle mass and function are expected to reverse cancer cachexia, exercise training could be beneficial for cancer cachexia therapy. Correlations between increased physical activity and improvements on muscle mass, function, and metabolism have been shown in some preclinical cancer models [118]. Other adjuncts such as nutritional support and anti-inflammatory treatments could also be included in cancer cachexia therapy. A multimodal rehabilitation to tackle different dimensions of cancer cachexia was proposed to manage cancer cachexia from various aspects [119]. 

As many of the studies referred to in this review were aimed at finding a correlation of biomarker levels with cancer cachexia, it is not surprising that there were discrepancies in cut-off values, even in cachexia found in the same type of cancer. Clear and reliable cut-off values are usually required for using biomarkers as diagnostic tools in the clinic. Hence, biomarkers with large variations between the cachectic and non-cachectic state are preferred. Although levels of some biomarker candidates showed statistical differences in cachectic and non-cachectic states, clear cut-off values could not be determined, as absolute differences or fold changes in these values were not prominent enough.

Another major concern is that none of these studies investigated the feasibility of using biomarkers to differentiate cancer cachexia from sarcopenia and anorexia, all conditions that cause muscle wasting and weight loss that can be found in patients with late-stage cancers. Since the treatments for these conditions are quite distinct, the biomarkers for cachexia should differentiate between these conditions. In this respect, tumor-derived biomarkers are preferred to biomarkers released by peripheral tissues, as their levels should not be affected by conditions such as sarcopenia and anorexia. Most factors investigated so far can be secreted from both tumor and host tissues, making it difficult to ascertain whether they are related to one cause of weight loss or another. Another approach may be the development of positive-negative diagnostic panels that can differentiate between cachexia, sarcopenia, and anorexia.

Detection methods for any potential biomarker should also be reliable and preferably subject to automation. Most circulating biomarkers are measured by enzyme-linked immunosorbent assay (ELISA). Accuracy and sensitivity of an ELISA are largely dependent on the specificity and concentration of antibodies used for the assay. In contrast, nucleic acid-based detection methods are more specific, sensitive, and cheaper than most protein-based methods. Hence, nucleic acid biomarkers are promising in this regard. Currently, most nucleic acid biomarkers are detected by qRT-PCR, which gives relative readings and requires controls for every test. In comparison, digital PCR is able to provide an absolute and ultrasensitive quantification of nucleic acid [120], which is the preferred method for practical use and could set a cut-off value for nucleic acid-based biomarker due to its absolute quantification.

**Figure 1:**
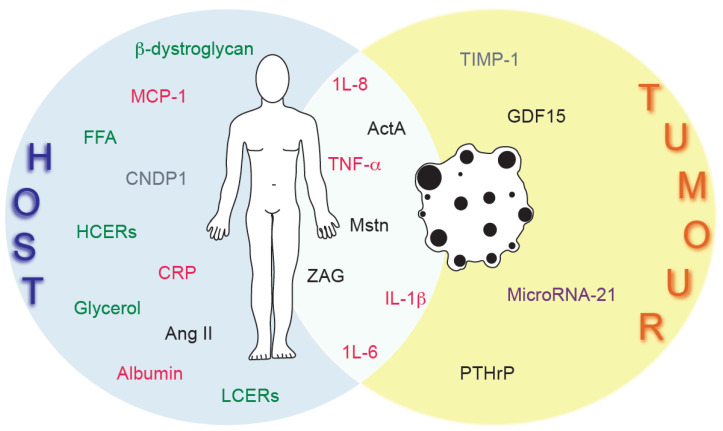
Sources of cancer cachexia biomarkers. Cancer cachexia biomarkers originate from the host, the tumor, or both. Cachexia inducing biomarkers are shown in black, inflammatory markers in red, muscle and fat wasting products in green, microRNAs in purple, and other factors in grey. MicroRNAs 203 and 130a are not included as the source is unknown.

In summary, there are no approved clinical biomarkers for cancer cachexia. This is currently a major impasse in the diagnosis and treatment of this condition, as cancer patients with increased muscle and fat loss have a definitively poor prognosis and do not tolerate conventional chemotherapeutic intervention. The ability to screen cancer patients with a panel of biomarkers (that cover a range of cancer types) that are upregulated in cachexia will allow clinicians to tailor treatment for the patient (Figure 1). Although there are currently no approved treatments for cancer cachexia itself, there are some promising targets and therapies in pre-clinical stages [30,121,122,123]. These raise the possibility that in combination with biomarker screening, traditional cancer treatment may be administered alongside potential cachexia therapies, increasing the strength of the patient and allowing them the time to undertake an entire course of treatment, thus enhancing survival rates.

## Figures and Tables

**Table 1:** Summary of cachexia-inducing factors as potential biomarkers of cancer cachexia.

Biomarker	Potential Source	Detection Method	Sample Format	Cancer Type	Median Level in Cachexia	Median Level in Non-Cachexia	Ref.
ActA	Tumor, host	ELISA	Serum	NSCLC, MPM	1.179 ng/mL	0.634 ng/mL	[26]
			Plasma	PDAC	1.997 ng/mL	1.027 ng/mL	[27]
				CRC, lung	0.558 ng/mL	0.397 ng/mL	[22]
Mstn	Tumor, host	ELISA	Plasma	CRC, lung	1.371 ng/mL	2.109 ng/mL	[22]
GDF15	Tumor	ELISA	Plasma	Lung	2 ng/mL	1 ng/mL	[33]
				Lung, GI	2.3 ng/mL	1.8 ng/mL	[32]
PTHrP	Tumor	IRMA	Serum	Lung, liver, PaCa, GI	5.7 pmol/L	Undetectable	[48]
		ELISA		NSCLC, CRC	205 pg/mL	Undetectable	[46]
ZAG	Tumor, host	ELISA	Serum	PaCa	40.3 µg/mL	28.9 µg/mL	[39]
Ang II	Host	ELISA	Plasma	PaCa, lung, breast	17 pg/mL	7.5 pg/mL	[49]

ELISA, enzyme-linked immunosorbent assay; IRMA, immunoradiometric assay; NSCLC, non-small-cell lung cancer; MPM, malignant pleural mesothelioma; CRC, colorectal cancer; PDAC, pancreatic ductal adenocarcinoma; PaCa, pancreatic cancer; GI, gastrointestinal cancer.

**Table 2:** Summary of inflammatory factors as potential biomarkers of cancer cachexia.

Biomarker	Potential Source	Detection Method	Sample Format	Cancer Type	Median Level in Cachexia	Median Level in Non-Cachexia	Ref.
TNF-α	Tumor, host	ELISA	Serum	PaCa	5.6 pg/mL	Undetectable	[56]
				GE, PaCa, CRC	15.9 pg/mL	12 pg/mL	[68]
			Plasma	GI	72.5 pg/mL	13.8 pg/mL	[60]
IL-6	Tumor, host	ELISA	Plasma	GI	160 pg/mL	30.3 pg/mL	[60]
		TIA	Serum	NSCLC	18 U/mL	2 U/mL	[59]
		ELISA	Plasma	Stomach, CRC	8.16 pg/mL	4.88 pg/mL	[73]
		ELISA	Serum	PaCa	207.8 pg/mL	162.3 pg/mL	[63]
IL-1β	Tumor, host	BioPlex cytokine assay	Plasma	GI, NSCLC	90.58 pg/mL	57.45 pg/mL	[62]
IL-8	Tumor, host	ELISA	Serum	PaCa	460.9 pg/mL	326.5 pg/mL	[63]
MCP-1	Host	ELISA	Plasma	PaCa	700 pg/mL	400 pg/mL	[11]
CRP	Host	ELISA	Serum	GI, PaCa, CRC	83 mg/L	4 mg/L	[68]
		ELISA	Plasma	GI	24.9 mg/L	14.9 mg/L	[60]
		Turbidimetric method	Plasma	GI, Lung	35 mg/L	17.6 mg/mL	[70]
Albumin	Host	NM	Plasma	GI, Lung	3.4 g/dL	3.8 g/dL	[70]

GE, gastroesophageal cancer; TIA, thymidine incorporation assay; NM, not mentioned.

**Table 3:** Summary of muscle and fat wasting products as potential biomarkers of cancer cachexia.

Biomarker	Potential Source	Detection Method	Sample Format	Cancer Type	Median Level in Cachexia	Median Level in Non-Cachexia	Ref.
β-dystroglycan	Host	WB	Muscle	GI	NA	NA	[69]
Glycerol	Host	NM	Plasma	GI	6.9 μmol·L^−1^·kg^−1^ fat	3.9 μmol·L^−1^·kg^−1^ fat	[76]
					6.2 μmol·L^−1^ ·kg^−1^ fat	3.1 μmol·L^−1^·kg^−1^ fat	[77]
					7.0 μmol·L^−1^·kg^−1^ fat	3.4 μmol·L^−1^·kg^−1^ fat	[78]
		Free Glycerol Reagent kit	Plasma	GI	4 µg/mL	3 µg/mL	[79]
FFA	Host	NM	Plasma	GI	53.8 μmol·L^−1^·kg^−1^ fat	32.5 μmol·L^−1^·kg^−1^ fat	[76]
					62 μmol·L^−1^·kg^−1^ fat	27 μmol·L^−1^·kg^−1^ fat	[77]
					80 μmol·L^−1^·kg^−1^ fat	40 μmol·L^−1^·kg^−1^ fat	[78]
HCERs	Host	MS	Plasma	GI	4 nmol/mL	3 nmol/mL	[13]
LCERs	Host	MS	Plasma	GI	4 nmol/mL	3.2 nmol/mL	[13]

NM, not mentioned; NA, not applicable; WB, western blot.

**Table 4:** Summary of microRNAs as potential biomarkers of cancer cachexia.

Biomarker	Potential Source	Detection Method	Sample Format	Cancer Type	Median Level in Cachexia	Median Level in Non-Cachexia	Ref.
MicroRNA-21	Tumor	qRT-PCR	Serum	CRC	NA	NA	[92]
MicroRNA-203	Unknown	qRT-PCR	Serum	CRC	NA	NA	[93]
MicroRNA-130a	Unknown	qRT-PCR	Plasma	HNC	NA	NA	[94]

HMC, head and neck cancer.

**Table 5:** Summary of other factors as potential biomarkers of cancer cachexia.

Biomarker	Potential Source	Detection Method	Sample Format	Cancer Type	Median Level in Cachexia	Median Level in Non-Cachexia	Ref.
CNDP1	Host	SBA, SIA	Plasma	GI	1500 MIF	2000 MIF	[98]
TIMP-1	Tumor	ELISA	Plasma	PDAC	860 ng/mL	550 ng/mL	[99]

SBA, sandwich bead arrays (SBA); SIA, sandwich immunoassays; MIF, mean intensity fluorescence.

## Abbreviations

ActA	Activin A
ActRIIB	Type IIB activin receptor
Ang II	Angiotensin II
Atrogin-1	Muscle Atrophy F-box gene
BAT	Brown adipose tissue
CNDP1	Carnosine dipeptidase 1
CRC	Colorectal cancer
CRP	C-reactive protein
ELISA	Enzyme-linked immunosorbent assay
FGF21	Fibroblast growth factor 21
GDF15	Growth/differentiation factor 15
GI	Gastrointestinal cancer
HCERs	Hexosyl-ceramides
HNC	Head and neck cancer
IRMA	Immunoradiometric assay
LBM	Lean body mass
LCERs	Lactosyl-ceramides
LLC	Lewis Lung Carcinoma
MCP-1	Monocyte chemoattractant protein-1
MPM	Malignant pleural mesothelioma
MS	Mass spectrometry
Mstn	Myostatin
MuRF1	Muscle RING-finger protein-1
NDPs	Neutrophil-derived proteases
NSCLC	Non–small-cell lung cancer
PaCa	Pancreatic cancer
PDAC	Pancreatic ductal adenocarcinoma
PPARγ	Peroxisome proliferator–activated receptor γ
PTHrP	Parathyroid Hormone release Peptide
qRT-PCR	Quantitative real-time reverse transcription PCR
TGF	Transforming growth factor
TIA	Thymidine Incorporation Assay
TIMP-1	Tissue inhibitor of metalloproteinases-1
WAT	White adipose tissue
ZAG	Zinc-α2-glycoprotein
